# Neuromodulation of inhibitory control using phase-lagged transcranial alternating current stimulation

**DOI:** 10.1186/s12984-024-01385-y

**Published:** 2024-05-30

**Authors:** Yukyung Kim, Je-Hyeop Lee, Je-Choon Park, Jeongwook Kwon, Hyoungkyu Kim, Jeehye Seo, Byoung-Kyong Min

**Affiliations:** 1 Department of Brain and Cognitive Engineering, Korea University, Seoul, 02841 Korea; 2 BK21 Four Institute of Precision Public Health, Korea University, Seoul, 02841 Korea; 3 Center for Neuroscience Imaging Research, Institute for Basic Science, Sungkyunkwan University, Suwon, 16419 Korea; 4 Institute of Brain and Cognitive Engineering, Korea University, Seoul, 02841 Korea

**Keywords:** EEG, Inhibitory control, Non-invasive neuromodulation, Phase-lagging, Transcranial alternating current stimulation

## Abstract

**Background:**

Transcranial alternating current stimulation (tACS) is a prominent non-invasive brain stimulation method for modulating neural oscillations and enhancing human cognitive function. This study aimed to investigate the effects of individualized theta tACS delivered in-phase and out-of-phase between the dorsal anterior cingulate cortex (dACC) and left dorsolateral prefrontal cortex (lDLPFC) during inhibitory control performance.

**Methods:**

The participants engaged in a Stroop task with phase-lagged theta tACS over individually optimized high-density electrode montages targeting the dACC and lDLPFC. We analyzed task performance, event-related potentials, and prestimulus electroencephalographic theta and alpha power.

**Results:**

We observed significantly reduced reaction times following out-of-phase tACS, accompanied by reduced frontocentral N1 and N2 amplitudes, enhanced parieto-occipital P1 amplitudes, and pronounced frontocentral late sustained potentials. Out-of-phase stimulation also resulted in significantly higher prestimulus frontocentral theta and alpha activity.

**Conclusions:**

These findings suggest that out-of-phase theta tACS potently modulates top-down inhibitory control, supporting the feasibility of phase-lagged tACS to enhance inhibitory control performance.

**Supplementary Information:**

The online version contains supplementary material available at 10.1186/s12984-024-01385-y.

## Background

Situations arise daily in which various features of our surroundings compete for attention and distract us from tasks. Cognitive control is a vital component of our daily lives, guiding our actions by promoting relevant information and suppressing irrelevant details or habitual behaviors. As cognitive control plays an important role in goal-directed behavior and is essential to effective functioning [1], techniques aimed at improving or enhancing cognitive control can provide valuable insight into and deepen our understanding of its underlying mechanisms and neural correlates. Although several studies have attempted to shed light on this topic [2–5], the neurophysiological rationales underlying these varying results remain unclear. This study aimed to provide neuromodulatory insight into the neurodynamics of cognitive control.

The Stroop task [6] is a well-established paradigm wherein cognitive inhibitory control distinguishes between intentional and automatic behaviors. For instance, in incongruent conditions, when word and text colors are conflicting, the color naming response time is longer than word reading, and color naming errors are more prevalent. This behavioral trend, referred to as the Stroop effect, implies that suppression of the automatic word-reading process is more challenging and requires inhibitory control for the color naming response [6, 7]. Functional neuroimaging studies uncovered numerous frontal brain regions that support cognitive control in the Stroop task, notably the left dorsolateral prefrontal cortex (lDLPFC) and the dorsal anterior cingulate cortex (dACC) [8–12]. The lDLPFC maintains a representation of task-related demands, regulates visual processes, and directs attention to relevant aspects of stimuli [9, 13–15]. Moreover, it plays a central role in exerting control over behavior, with extensive connections to the sensory and motor areas [1, 16]. The dACC is also associated with attentional processes, including error detection, conflict monitoring, and performance evaluation [17–19]. Activity in this region closely mirrors the level of control recruited in conflict scenarios and has a strong functional relationship with the lateral prefrontal cortex [20–22].

**Fig. 1:**
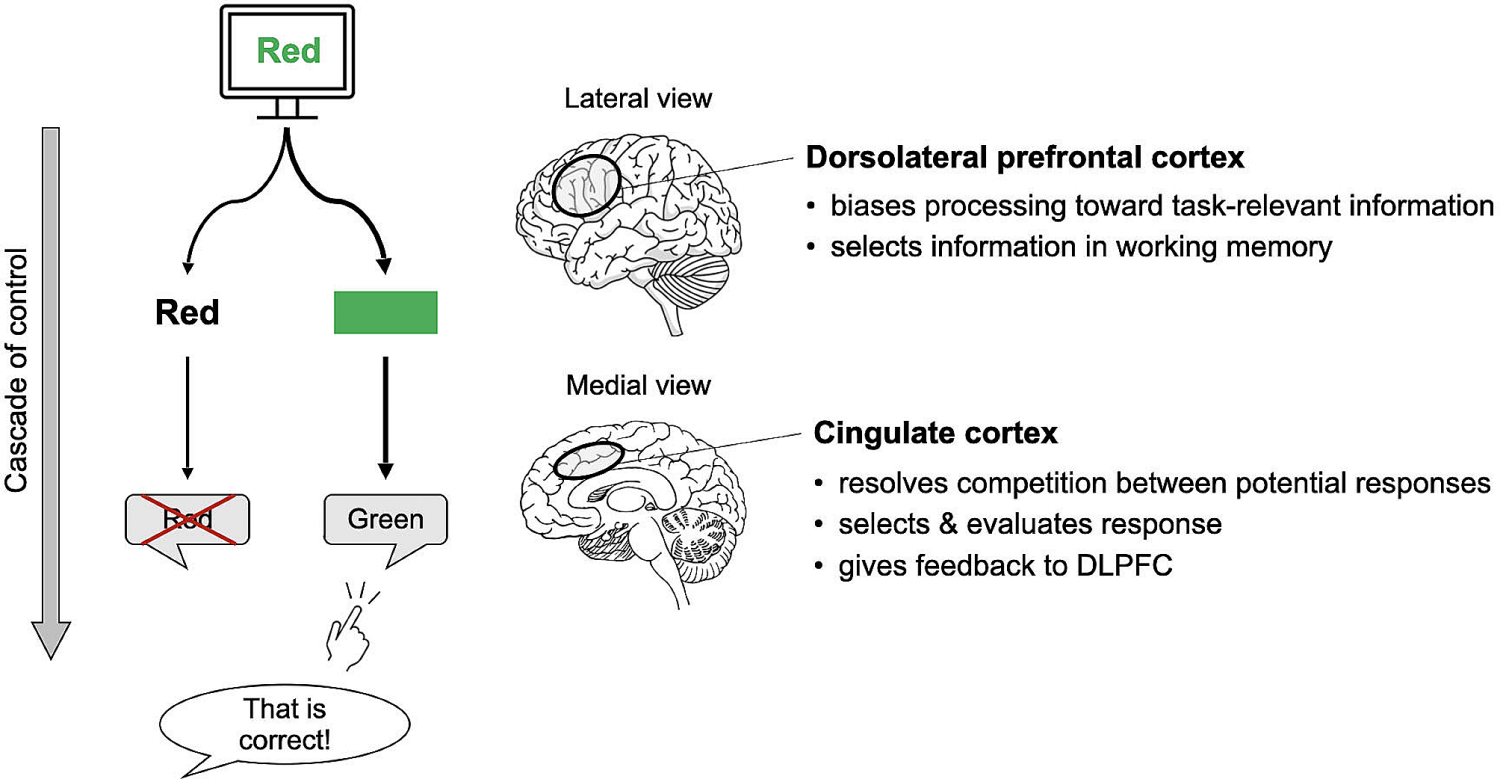
“Cascade of control” model of the Stroop effect (adapted from [27]). DLPFC, dorsolateral prefrontal cortex

The most basic computational model explaining control processes in the Stroop task uses two competing pathways: automatic (encoding a word feature) and control-demanding (encoding a color feature) processes [23]. This model can be expanded to incorporate a conflict-monitoring unit represented by the dACC, which signals the DLPFC-mediated cognitive control required to facilitate processing in the task-relevant pathway [11, 18]. Recent models further explored and tested the temporal dynamics between the two regions [24–27]. For instance, the “cascade of control” model [27] proposes that control mechanisms intervene at various task performance stages to resolve the Stroop conflict effect (Fig. 1).

The interaction between the lDLPFC and the dACC can also be investigated electrophysiologically. The two regions exhibit stronger connectivity in conflict situations such as the incongruent condition in the Stroop task, particularly in the theta frequency band (4–8 Hz) [26, 28–30]. Furthermore, increased theta power has been observed in the midfrontal regions after events that require greater control [31–33]. These theta dynamics play a crucial role in coordinating diverse brain regions and orchestrating the temporal flow of midfrontal neuronal processes, thus enabling executive conflict control functions [30, 33]. Studies conducted in conjunction with functional imaging methods or involving source reconstruction suggested that the medial prefrontal cortex generates this oscillatory activity [28, 31, 34, 35]. This activity is often accompanied by signature event-related potential (ERP) components such as N2 and late sustained potentials (LSP) [36, 37].

On the other hand, non-invasive neuromodulation of cognitive control processes may impact cognitive performance. Previous studies have used various non-invasive brain stimulation methodologies such as transcranial direct current stimulation (tDCS), transcranial alternating current stimulation (tACS), and transcranial magnetic stimulation (TMS) [38–41]. Several studies have explored the causal influence of specific brain oscillations on cognitive function [42–44]. By employing these approaches and manipulating specific stimulation parameters, researchers have successfully demonstrated the intentional modulation of brain activity as well as improved performance in cognitive tasks, such as working memory performance [45–48] or inhibitory control [49, 50]. For example, neuronal communication depends on the coherent oscillation of activated neuronal groups, which enables effective interactions through synchronized communication windows and supports cognitive flexibility [51]. By using tACS to synchronize intrinsic neuronal oscillations to the applied stimulation phase, brain rhythms can be effectively entrained through phase specificity [52, 53]. Phase synchronization is an essential neuronal mechanism that manages intrinsic communication between distinct nodes to improve cognitive functions such as executive skills, attention, and context processing [54–56]. Accordingly, in-phase tACS seeks to enhance synchronization and coordination between brain regions to improve specific cognitive functions, whereas out-of-phase tACS aims to introduce interference or desynchronization to modulate cognitive processes [57–61]. Nevertheless, the specific type of phase lag that can effectively enhance task performance and the underlying neurophysiological mechanisms remain unclear.

Thus, here we designed a paradigm to investigate whether theta-frequency tACS administered with different relative phase lags between the dACC and the lDLPFC affects cognitive control performance. It has been reported that the influence of lDLPFC on Stroop interference is mediated by later dACC activity [25], consistent with the temporal course hypothesis posited by the “cascade of control” model [27]. The different phase lags simulate temporally delayed stimulation between the lDLPFC and the dACC. We used the color-word Stroop task [6] to examine the effects of tACS on inhibitory function and executive (top-down) control. Individual theta peak frequencies were employed, and the dACC and lDLPFC were stimulated in-phase (0° relative phase lag) or out-of-phase (180° relative phase lag). We hypothesized that out-of-phase (phase-lagged) tACS would effectively modulate inhibitory task performance. Owing to highly contaminated noise in the EEG data that is created by simultaneous tACS currents, the EEG data were obtained during the Stroop task after each tACS session was completed.

## Methods

### Participants

Twenty-four healthy volunteers (mean age, 23.67 ± 0.53 years; 13 men, 11 women) participated in this study. All participants were right-handed and had normal or corrected-to-normal vision. None of the participants reported having a history of psychiatric or neurological disorders, and no color blindness was determined using the Ishihara color test. All participants were free of contraindications to magnetic resonance imaging (MRI) scanning. Written informed consent was obtained from all participants. This study was conducted in accordance with the ethical guidelines of the Korea University Institutional Review Board (KUIRB-2021-0209-08).

### Task paradigm and experimental procedure

**Fig. 2:**
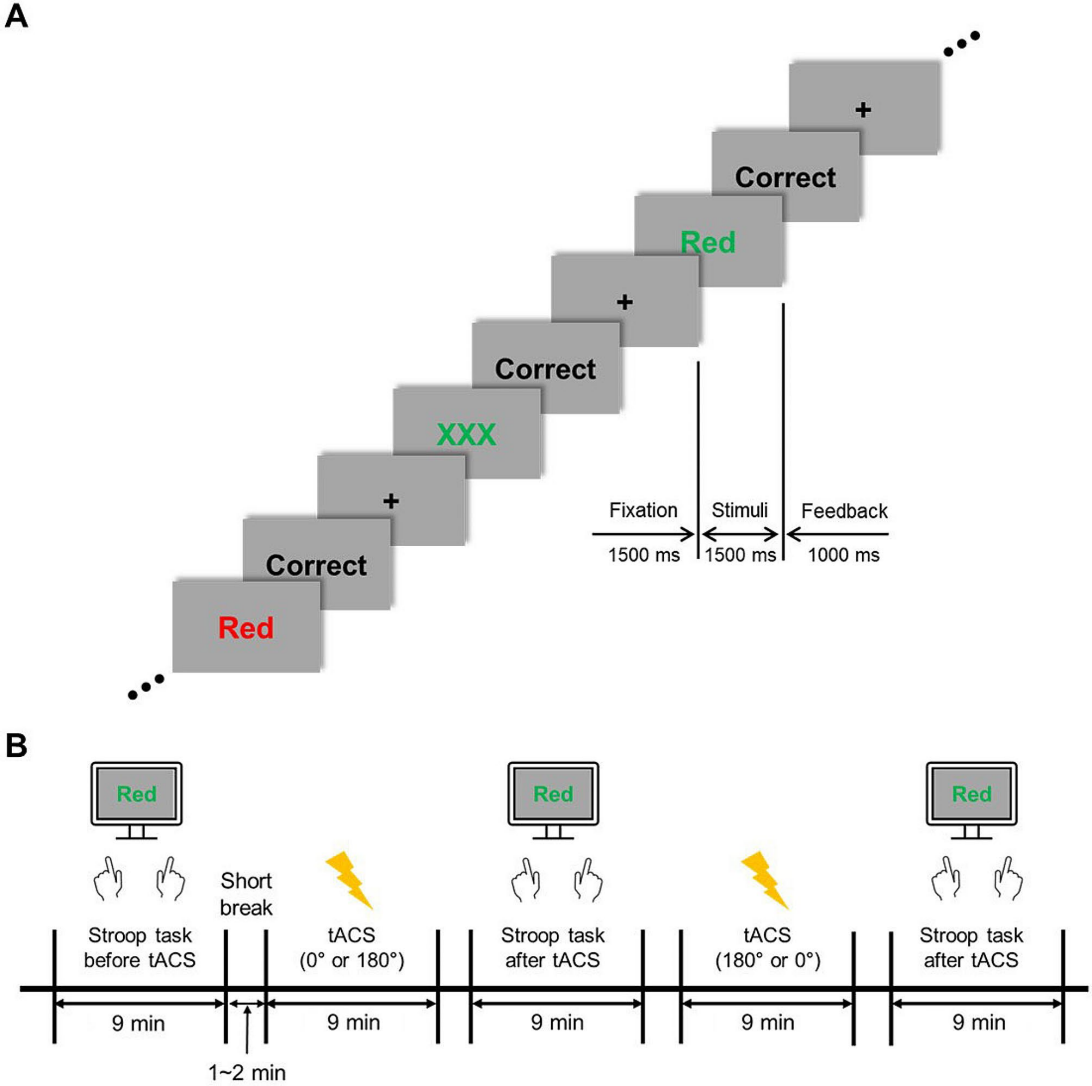
Experimental procedure and the Stroop task. (**A**) The Stroop task paradigm. (**B**) Time flow of the experimental sessions. Each stimulation session was followed by the task session. The order of the stimulation sessions (in-phase/out-of-phase or out-of-phase/in-phase) was counterbalanced across the participants. tACS, transcranial alternating current stimulation

We used the color-word Stroop task [6] to investigate inhibitory control functions (Fig. 2A). The Stroop task is suitable for studying neural activity related to cognitive control since it requires attentional allocation and inhibitory processes with conflicting features. The task stimuli comprised congruent, neutral, and incongruent conditions. The congruent and incongruent stimuli were color words (“Red” and “Green”) written in congruent or incongruent colors, while neutral stimuli were meaningless streams of letters (“XXX”). The items were presented randomly and equally to each participant using presentation software (E-prime 3.0 Professional, Psychology Software Tools, USA). The subtended visual angle for each item was set at 5°. The participants were instructed to respond as quickly as possible by pressing a button with their right or left index finger indicating the color of the presented letter stimulus. The response hands were counterbalanced across the participants. As depicted in Fig. 2A, a fixation cross was presented at the center of the screen for 1500 ms, followed by presentation of the stimulus for 1500 ms. During this time, the participants’ responses were recorded. For an additional 1000 ms, visual feedback in the form of “Correct,” “Incorrect,” or “No Response” was displayed on the monitor to motivate the participants and encourage improved performance of the task. Each participant completed three task sessions, each comprising 45 trials per congruency condition for a total of 135 trials per task session. The initial task session was used to determine the individual theta peak frequencies. The individual theta frequencies for each participant were determined as the dominant theta peak frequency during the Stroop task performance. Based on the EEG data from the first experimental session without tACS treatment (Fig. 2B), the theta frequencies (4–8 Hz) for each participant were individually determined and were administered as personalized theta-frequency sinusoidal tACS signals. This approach is based on the close relationship between the midfrontal theta frequency band during conflict situations and the central executive function, as demonstrated in previous research [31, 33]. Before the main tACS experiment was performed, the midfrontal theta peak frequencies were individually determined using the fast Fourier transform (without padding) of artifact-free EEG trials. The frequency (ranging from 4 to 8 Hz) was selected when its maximum power at the midfrontal electrodes Fz, F1, and F2 was detected within the time window between stimulus onset and 1 s poststimulus during the no-tACS Stroop task (mean theta peak frequency: 5.79 ± 1.61 Hz).

**Fig. 3:**
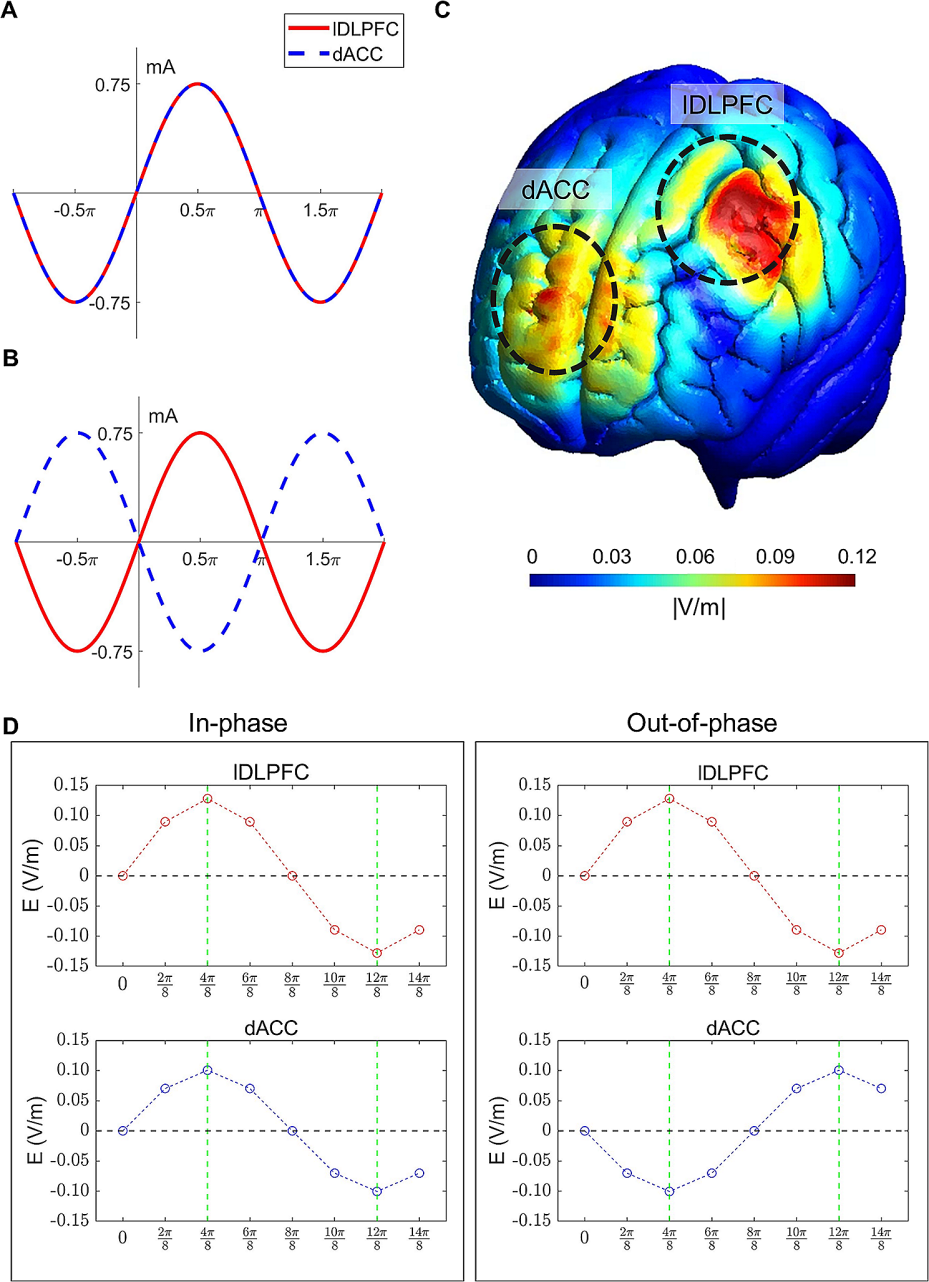
Stimulation protocol for in-phase and out-of-phase tACS. (**A**) In-phase stimulation waveforms to the lDLPFC (red solid line) and dACC (blue dashed line). (**B**) Out-of-phase stimulation waveforms to the lDLPFC (solid red line) and dACC (dashed blue line). (**C**) A sample simulation of the tACS-induced electric field at the lDLPFC and dACC. The unit *|*V/m*|* denotes the normalized strength of the induced electric field. (**D**) The simulated electric intensity (V/m) of each phase bin (eight bins for $2{\pi }$—that is, by a step of $\frac{2{\pi }}{8}$) is plotted in the lDLPFC (two upper plots in red) and in the dACC (two lower plots in blue; a left panel for the 0°-phase-lag and a right panel for the 180°-phase-lag tACS condition). Note that the phase of the lDLPFC stimulus advanced that of the dACC stimulus by approximately 180° (vertical green dashed lines indicate peak phases of the lDLPFC and dACC). dACC, dorsal anterior cingulate cortex; lDLPFC, left dorsolateral prefrontal cortex

Each experiment consisted of five 9-min-long sessions, between which short breaks were given (Fig. 2B). We conducted two stimulation sessions, during which tACS was applied for 9 min with a 0° (in-phase) or 180° (out-of-phase) phase difference between the lDLPFC and dACC (Fig. 3). The order of the stimulation sessions was counterbalanced across the participants. Each stimulation session was followed by the performance of a Stroop task. The participants were debriefed immediately after the last task session, during which they indicated their subjective perception (or any uncomfortable experience, including retinal phosphenes) of the stimulation. Eventually, 21 of the 24 participants did not report irritation induced by the tACS treatment. One individual experienced dizziness, another reported sore eyes, and the remaining participant reported a migraine-like feeling at times.

### Target localization using fMRI

Before the main experiment (with a minimum interval of two days preceding the main experiment), we performed functional MRI (fMRI) to individually localize the task-relevant brain regions that were activated during the Stroop task and to optimize the stimulation electrode placement. We used the initial fMRI data during the Stroop task without tACS to individually identify the target regions, specifically the lDLPFC and dACC. Throughout the Stroop task, whole-brain images were acquired using a 32-channel head coil within a 3T MAGNETOM Trio Tim Syngo scanner (Siemens Healthcare, Erlangen, Germany). A total of 270 blood oxygenation level–dependent (BOLD) fMRI image volumes were acquired using an interleaved T2*-weighted echo-planar imaging sequence with the following parameters: repetition time (TR), 2000 ms; echo time (TE), 30 ms; flip angle (FA), 90°; multi-band acceleration factor, 3; acquisition matrix, 96 × 96; field of view, 192 × 192 mm^2^; in-plane voxel size, 2 × 2 × 2 mm^3^; and no slice gap. High-resolution structural scans of three-dimensional anatomical magnetization prepared rapid acquisition gradient echo images were obtained for each subject after the fMRI data collection (TR, 2.3 s; TE, 2.13 ms; inversion time, 0.9 s; FA, 9°; acquisition matrix, 256 × 256; in-plane voxel size, 1 × 1 × 1 mm^3^; 224 sagittal slices).

**Fig. 4:**
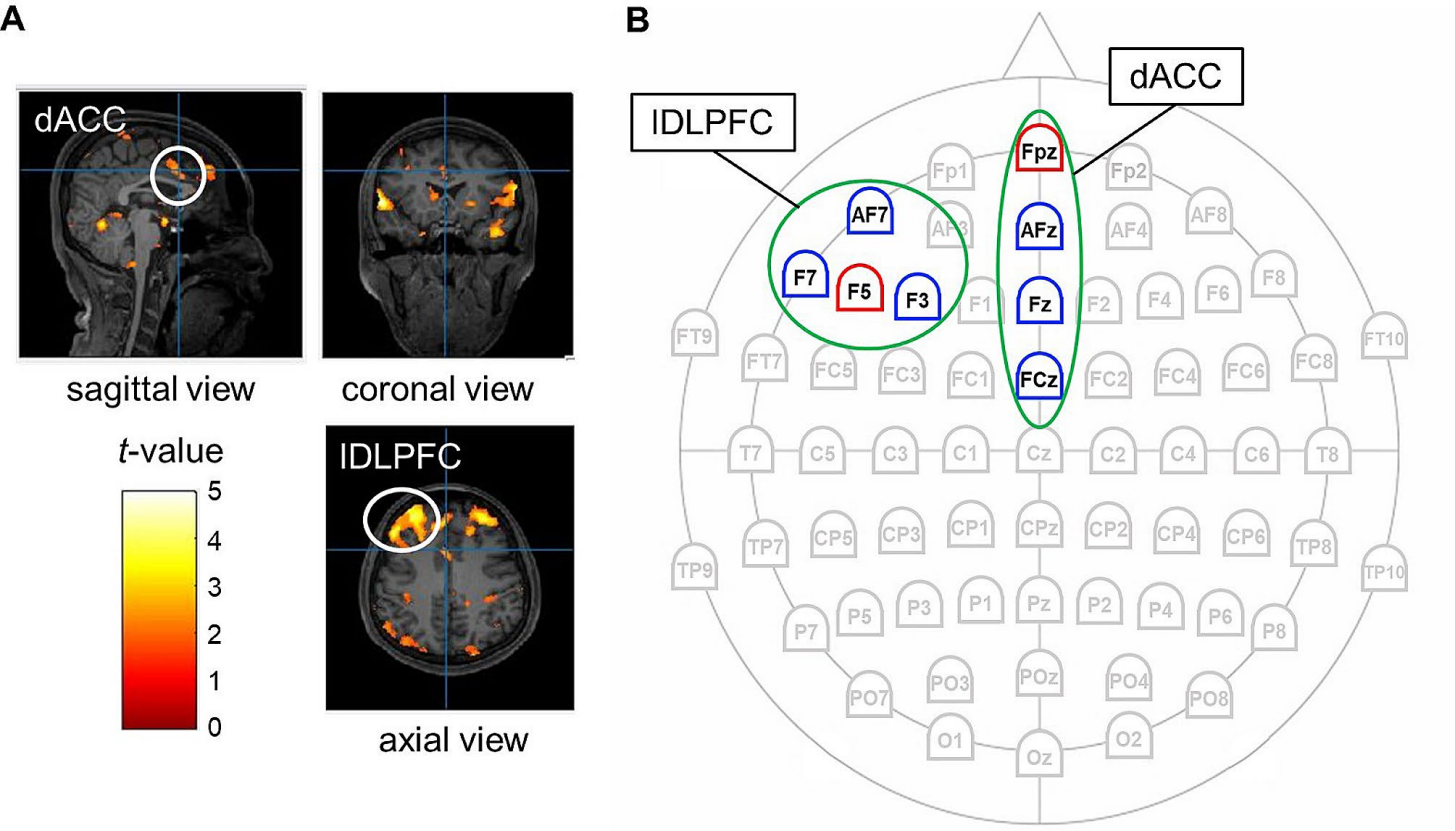
Target-region identification and electrode-placement montage. (**A**) An example of target-region identification using individual functional magnetic resonance imaging data. (**B**) A sample montage of the optimized electrode placements used for high-definition transcranial alternating current stimulation. The stimulation input electrode for each region of interest (highlighted in green) is marked in red, and the three return electrodes are marked in blue. The montages of all participants are provided in Supplementary Figure S1. dACC, dorsal anterior cingulate cortex; lDLPFC, left dorsolateral prefrontal cortex

Following the preprocessing of BOLD images through the standard task-based fMRI pipeline (slice-timing correction, motion correction, co-registration, grey/white matter segmentation, normalization, and spatial smoothing using a 6-mm full-width at half-maximum Gaussian kernel) with Statistical Parametric Mapping (SPM12; https://www.fil.ion.ucl.ac.uk/spm/software/spm12) in MATLAB software (R2021a; MathWorks, USA), we analyzed whole-brain activity for each trial type (congruent, incongruent, and neutral) relative to a fixation block for each participant. Subject-specific optimized coordinates of the stimulated target regions, specifically the lDLPFC and the dACC, were co-registered on individual T1 images to ensure a spatially accurate stimulation. A sample montage for a single individual is shown in Fig. 4A. Generally, an input-return module configuration is used to coordinate the stimulation and return channels (i.e., return electrodes are arranged around the stimulation electrode). The currents of all stimulation and return channels were set to maintain the total amount of current at 0.

### EEG acquisition and tACS stimulation

Before the electroencephalography (EEG) experiment, we conducted an fMRI test to localize the brain regions activated during the Stroop task and to optimize the stimulation electrode placement. We used fMRI data from each participant to identify the target regions, specifically the lDLPFC and the dACC. The electrode placement was then planned to maximize the tACS-induced electric field intensity in the target regions. Optimization of electrode placement was performed using SimNIBS (ver. 3.2.6, DRCMR & DTU, Denmark) [62] and tES LAB (ver. 3.0, NeuroPhet, Seoul, Korea). To ensure precise, spatially accurate stimulation, participant-specific optimized coordinates of the target regions for stimulation were calculated based on individual T1 images that were obtained prior to the main experiment. Electrode placements were selected to optimize the strength of the electric field at the target locations. The optimal stimulation power was determined based on the attainment of maximum intensity at both target regions (i.e., the lDLPFC and dACC) when manipulating the combination of input/return electrode positions around the scalp of the target regions using the individual sensation thresholds of the input current. For each target region, we selected one stimulation electrode (marked in red) and three return electrodes (marked in blue; Fig. 4B).

High-definition tACS was administered using a 65-channel high-definition transcranial electrical stimulation (HD-tES) device (M×N 65 HD-tES; Soterix Medical Inc., USA), with individually customized frequency and intensity for each participant. Ag/AgCl sintered ring electrodes (HD-Electrode, Soterix Medical Inc., USA; surface area: 1.13 cm^2^) were used for stimulation and affixed to the scalp using an electrolyte medium (HD-GEL, Soterix Medical Inc., USA) in an EEG cap (actiCAP, Brain Products GmbH, Germany). Prior to the stimulation experiment, the impedances of the stimulation electrodes were maintained below 30 kΩ. Regarding the individually applied stimulation amplitude, the mean amplitude was 0.86 ± 0.33 mA. Since the lDLPFC and dACC exhibit stronger connectivity in conflict situations, such as the incongruent condition in the Stroop task, particularly in the EEG theta band [26, 28–30], and the neuromodulatory effect of resonating the brain oscillations responsible for Stroop task performance would be maximized when the stimulus wave (i.e., tACS) resembles the target wave (i.e., human brain wave) as closely as possible, the theta frequency was used for the tACS frequency in the present study. The theta frequencies (4–8 Hz) for each participant were individually determined based on the first experimental session and administered as personalized theta-frequency sinusoidal waves. This approach is based on the close relationship between the midfrontal theta frequency band during conflict situations and central executive function [31, 33]. We adjusted the stimulation intensity individually for each participant in a stepwise manner to ensure that it was below the individual sensation threshold and that the total stimulation intensity did not exceed 1.5 mA.

A simulation program (tES LAB software, ver. 3.0, NeuroPhet, Korea) was used to examine whether the stimulation signals matched the intended phase lag (0° or 180°) before the main study. The mean simulation electric-field intensity was 0.11 ± 0.02 V/m at the activated cortical region. As shown in Fig. 3C, the simulation results demonstrated that the activation regions were well aligned with the intended target regions. Moreover, we investigated whether the phase difference between the lDLPFC and the dACC stimulation signals exactly matched the intended phase lag (0° or 180°; Fig. 3D).

EEG signals were recorded using a BrainAmp DC amplifier (Brain Products, Germany) with 64 Ag/AgCl electrodes, according to the international 10–10 system. A reference electrode was placed at the tip of the nose and the AFz electrode was used as the ground electrode. Electrode input impedances were kept below 10 kΩ prior to the data acquisition. The sampling rate was set at 500 Hz.

### Data analysis

We analyzed both reaction times and accuracy as behavioral measures of task performance. Each participant’s reaction times were fitted to a gamma distribution and collected within a 95% confidence interval [63]. For the subsequent EEG analyses, only correct response trials were selected. Preprocessing of the electrophysiological data was performed offline using the EEGLAB toolbox [64]. We applied a 0.5-Hz high-pass filter and a 60-Hz notch filter to the raw EEG signals. Eye and muscle artifacts were automatically identified with an 80% threshold (probability, 0.8) and removed from the data using independent component analysis and ICLabel, an automated independent component classifier [65, 66]. The EEG data were segmented from the 1500 ms prestimulus to the 2500 ms poststimulus, with a total of 4000 ms for each epoch. After epoching the EEG data, demeaning and detrending were performed. Subsequent analyses were performed using MATLAB software (R2022b; MathWorks, USA).

To investigate the ERP components, we further filtered the EEG signals from 1 to 30 Hz. The averaged signals were baseline-corrected using a 200-ms time window before stimulus presentation. We inspected the tACS-mediated ERP alterations during the Stroop task. Time ranges were determined based on previous ERP studies on cognitive control [36, 37] and adjusted according to the grand averages with individual variations, while considering the time windows of adjacent ERP components. N1, P1, and N2 were designated as early ERP components, whereas LSP was considered a late ERP component. Electrodes for the ERP analyses were selected based on the regions of the brain in which the activity was most prominent. For the frontocentral N1 component, negative peak amplitudes were detected within a time window of 30–130 ms for the FCz, FC1, FC2, Cz, C1, C2, CPz, CP1, and CP2 electrodes. For the parieto-occipital P1 component, positive peak amplitudes were detected within a time window of 20–120 ms for the O1, O2, PO7, and PO8 electrodes. For the frontocentral N2 component, negative peak amplitudes were detected within a time window of 150–250 ms for the Fz, F1, F2, FCz, FC1, FC2, Cz, C1, and C2 electrodes. For the frontocentral LSP, the mean amplitudes were detected within a time window of 550–800 ms for the Fz, F1, F2, FCz, FC1, FC2, Cz, C1, and C2 electrodes.

To investigate EEG oscillatory activity, we performed a complex Morlet wavelet convolution of the EEG signals. We employed a wavelet family with a constant ratio of seven cycles [67] and frequencies of 1–15 Hz. A wavelet transform was conducted for each trial, and the absolute values of the resulting transforms were averaged. This measure of signal amplitude in single trials reflects the total activity for a certain frequency range. As prestimulus theta and alpha activity are known indicators of top-down preparation [68, 69], we investigated theta (4–8 Hz) and alpha (8–13 Hz) power before the stimulus presentation. Electrodes for the spectral analyses were selected based on the task-relevant topographic distribution, and the analytic time windows were selected based on the smearing effects of the wavelet analysis and the effective numbers of wavelengths of the analytic frequencies. For the prestimulus frontocentral theta activity, theta power was computed in an interval of 200–500 ms before stimulus onset and averaged across the Fz, F1, F2, and FCz electrodes. For prestimulus frontocentral and parieto-occipital alpha activity, alpha power was computed in a time window of 100–400 ms before stimulus onset and averaged across the Fz, F1, F2, and FCz, as well as across the POz, PO3, and PO4 electrodes, respectively. The Shapiro–Wilk test revealed that the data was not normally distributed. Therefore, the Wilcoxon signed-rank test, a non-parametric approach, was employed in this study. Behavioral data, ERP components, and EEG spectral power were compared between the in-phase and out-of-phase stimulation conditions within each congruency condition. A statistical power analysis [70] for the Wilcoxon signed-rank test estimated the minimum number of samples, ≥ 15 (24 participants in the present study), with an effective size = 0.8, statistical power (1-𝛽) = 0.8, and ɑ = 0.05.

## Results

### Behavioral data

**Fig. 5:**
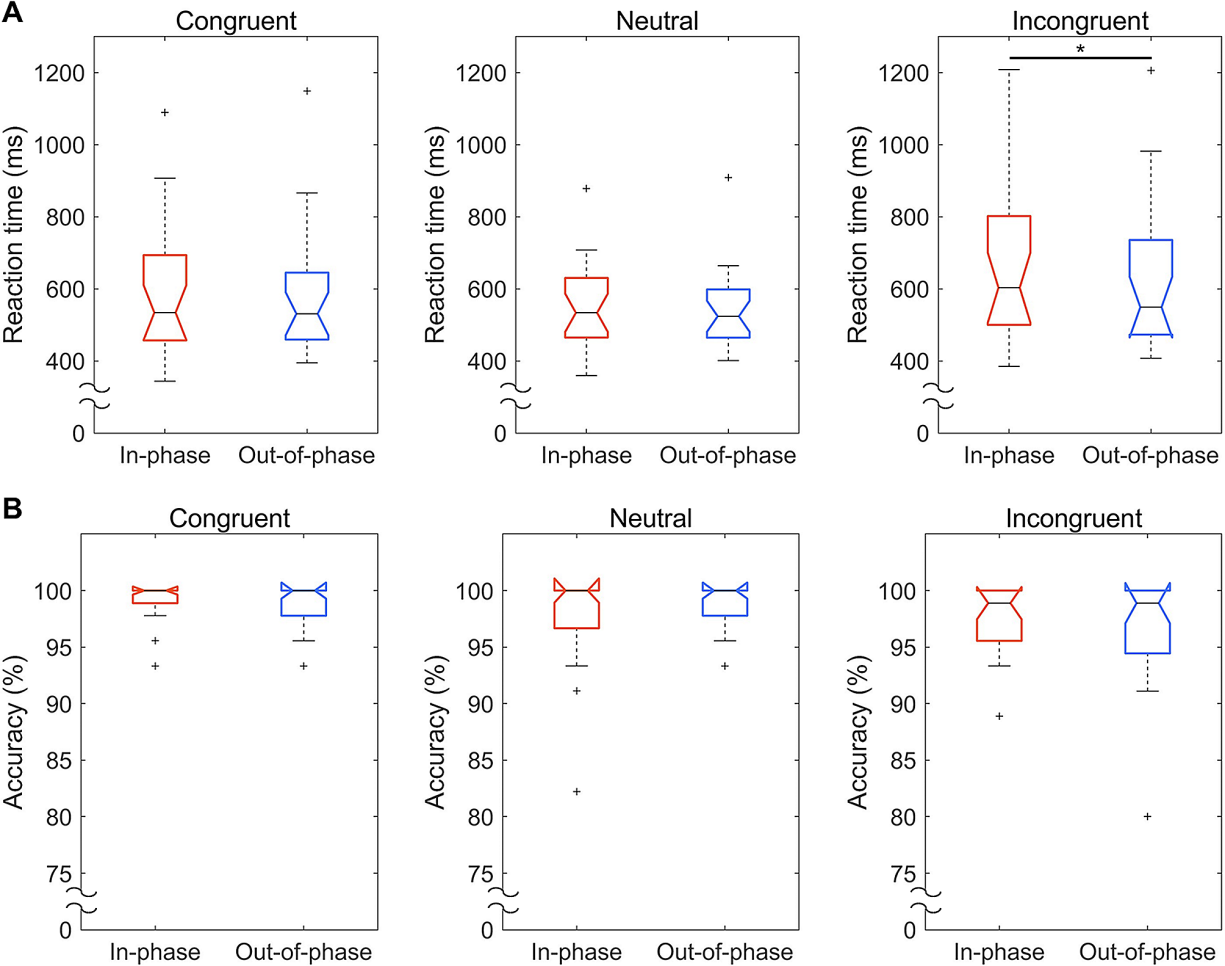
Phase-dependent tACS-mediated changes in reaction times and accuracies. (**A**) Reaction times following in-phase (red) and out-of-phase (blue) stimulation. (**B**) Task performance accuracies following in-phase (red) and out-of-phase (blue) stimulation. In the box plots, boxes are drawn from the first to the third quartile. The horizontal lines within boxes denote the median, and the whiskers extend from each quartile to the minimum or maximum with excluded outliers marked as small crosses. The asterisk represents statistical significance (*p < 0.05)

In the incongruent condition, out-of-phase stimulation exhibited significantly reduced reaction times compared to in-phase stimulation (*Z* = 2.49, *p* < 0.05; in-phase, 603.26 ms; out-of-phase, 549.17 ms; Fig. 5A). The differences in reaction times between in-phase and out-of-phase tACS in the congruent (*Z* = 0.66, *n.s.*) and neutral (*Z* = 0.63, *n.s.*) conditions were not statistically significant. There were no significant differences between the stimulation conditions in terms of task performance accuracy for the congruent (*Z* = 1.57, *n.s.*), neutral (*Z* = − 1.11, *n.s.*), and incongruent (*Z* = 1.25, *n.s.*) conditions (Fig. 5B).

### ERP components

**Fig. 6:**
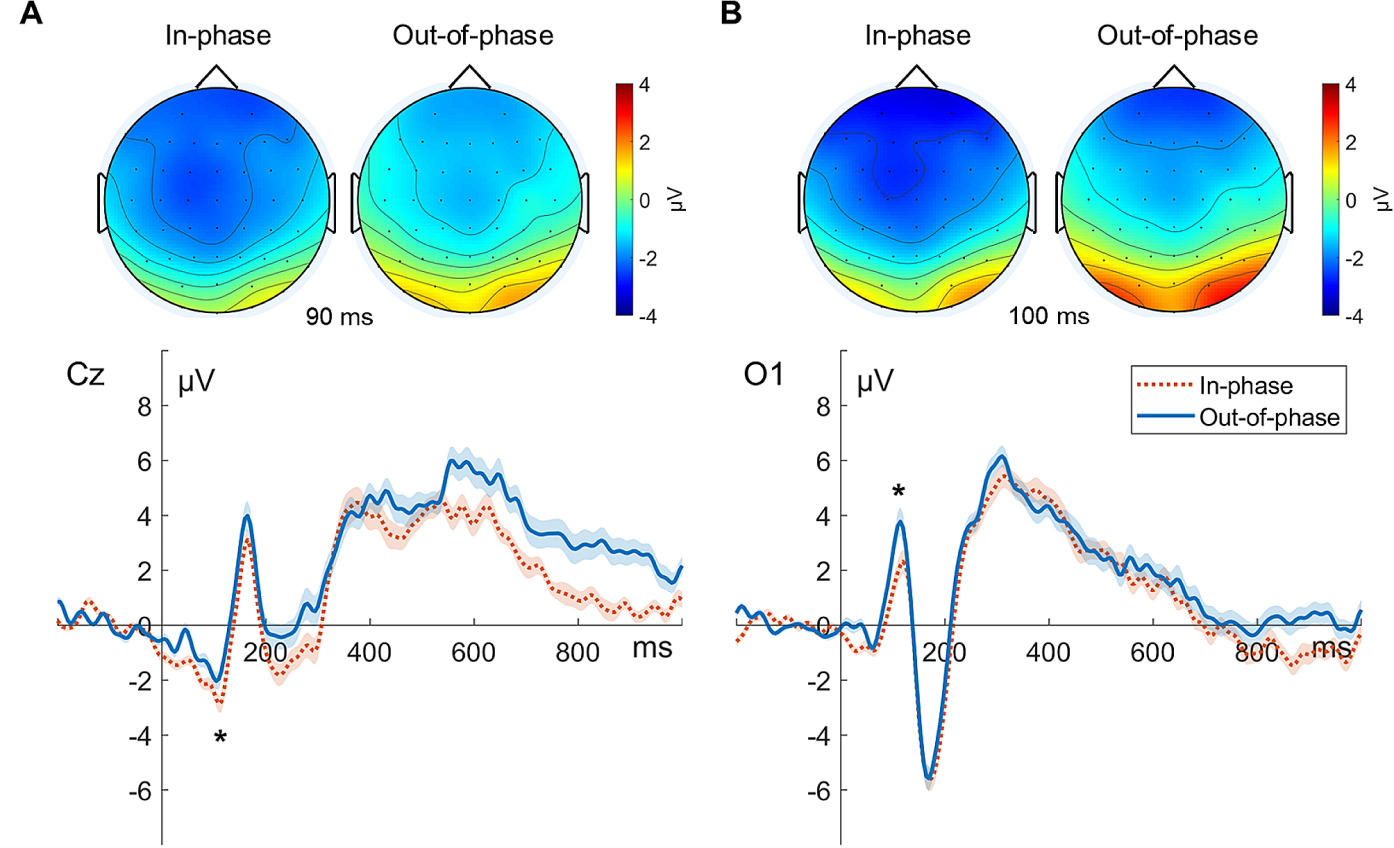
Phase-dependent tACS-mediated topographical maps and time courses of N1 and P1 components in the incongruent condition. (**A**) The upper panel illustrates the grand-averaged topographical distributions for the N1 component (at 90 ms poststimulus). The lower panel shows the grand-averaged ERP time courses for in-phase (orange dotted line) and out-of-phase (blue solid line) stimulation at electrode Cz. (**B**) The upper panel illustrates the grand-averaged topographical distributions for the P1 component (at 100 ms poststimulus). The lower panel shows the grand-averaged ERP time courses for in-phase (orange dotted line) and out-of-phase (blue solid line) stimulation at the O1 electrode. Topographies are displayed in the order of in-phase (left) and out-of-phase (right) tACS. The view of the topography is from the vertex perspective with the nose at the top of the image. For ERP time courses, time zero indicates stimulus onset. The error bands indicate the standard errors of the mean, and the asterisks represent statistical significance (*p < 0.05)

For the N1 component, we found significantly reduced N1 amplitudes in the out-of-phase versus in-phase stimulation condition in the incongruent (*Z* = − 2.31, *p* < 0.05; in-phase, − 4.35 µV; out-of-phase, − 3.11 µV) and neutral (*Z* = − 2.57, *p* < 0.05; in-phase, − 4.42 µV; out-of-phase, − 3.18 µV) conditions. The differences in the congruent condition were not statistically significant (*Z* = − 0.51, *n.s.*). Regarding the parieto-occipital P1 component, we observed significantly enhanced P1 amplitudes in the out-of-phase versus in-phase stimulation condition in the incongruent condition (*Z* = − 2.11, *p* < 0.05; in-phase, 2.49 µV; out-of-phase, 3.97 µV). The differences in P1 amplitudes between them in the congruent (*Z* = − 0.94, *n.s.*) and neutral (*Z* = − 1.94, *n.s.*) conditions were not statistically significant (Fig. 6).

**Fig. 7:**
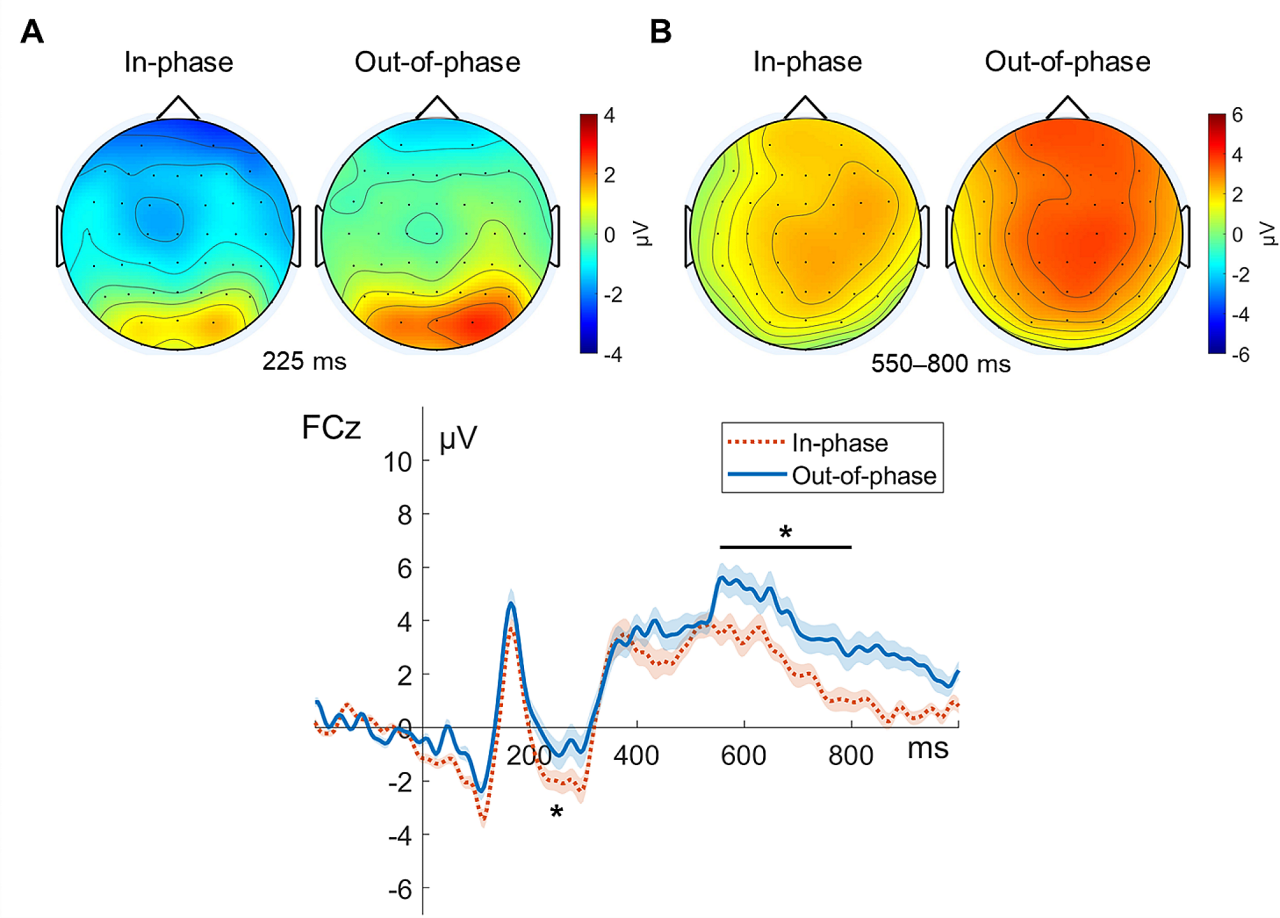
Phase-dependent tACS-mediated topographical maps and time courses of N2 and LSP in the incongruent condition. The upper panel illustrates the grand-averaged topographical distributions for the (**A**) N2 (at 225 ms poststimulus) and (**B**) LSP (averaged over 550 to 800 ms poststimulus). The lower panel shows the grand-averaged ERP time courses for the in-phase (orange dotted line) and out-of-phase (blue solid line) stimulation at the electrode FCz. Topographies are displayed in the order of in-phase (left) and out-of-phase (right) tACS. The view of the topography is from the vertex, with the nose at the top of the image. For ERP time courses, time zero indicates stimulus onset. The error bands indicate the standard errors of the mean, and the asterisks represent statistical significance (*p < 0.05)

In addition, significantly reduced frontocentral N2 amplitudes were detected in the out-of-phase versus in-phase stimulation condition in the incongruent condition (*Z* = − 2.31, *p* < 0.05; in-phase, − 3.02 µV; out-of-phase, − 1.37 µV). The differences in the N2 amplitudes between them in the congruent (*Z* = 0.63, *n.s.*) and neutral (*Z* = − 1.20, *n.s.*) conditions did not reach statistical significance. Finally, for the frontocentral LSP, the mean LSP amplitudes were significantly higher in the out-of-phase versus in-phase stimulation condition in the incongruent condition (*Z* = − 1.97, *p* < 0.05; in-phase, 2.79 µV; out-of-phase, 4.31 µV). The differences between them in the congruent (*Z* = − 1.86, *n.s.*) and neutral (*Z* = − 1.20, *n.s.*) conditions were not statistically significant (Fig. 7).

### EEG spectral power

**Fig. 8:**
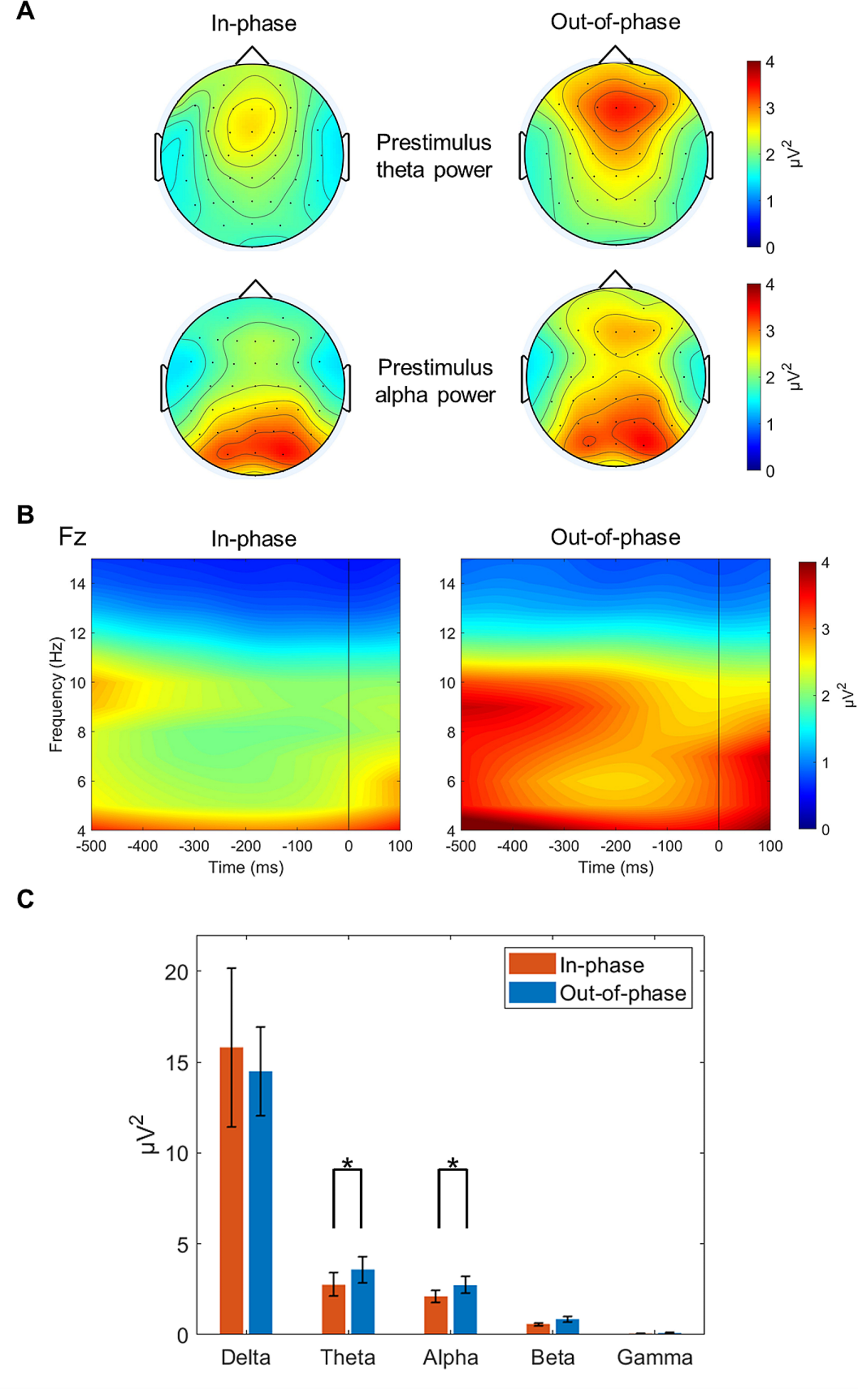
Phase-dependent tACS-mediated topographical maps of prestimulus theta and alpha power in the incongruent condition. (**A**) The topographical maps show the grand-averaged prestimulus theta (500 to 200 ms prestimulus) and alpha (400 to 100 ms prestimulus) power distributions given in the order of in-phase (left) and out-of-phase (right) stimulation. The view of the topography is from the vertex perspective with the nose at the top of the image. (**B**) The time-frequency plots represent the spectral power of total activity at the electrode Fz. Time 0 indicates stimulus onset. The color bar indicates the power (µV^2^). (**C**) Comparison of prestimulus spectral power (µV^2^; in the frontocentral region averaged across Fz, F1, F2, and FCz, from 400 to 200 ms prestimulus) between in-phase (red bars) and out-of-phase (blue bars) tACS treatments across the delta (1–4 Hz), theta (4–8 Hz), alpha (8–13 Hz), beta (13–30 Hz), and gamma (30–50 Hz) bands. The error bars indicate the standard errors of the mean, and the asterisks represent statistical significance (*p < 0.05). Note the phase-dependent tACS-mediated significant differences particularly in theta and alpha bands

Regarding the prestimulus frontocentral theta activity, significantly enhanced theta power was observed in the out-of-phase versus in-phase stimulation condition in the congruent (*Z* = − 2.43, *p* < 0.05; in-phase, 2.84 µV^2^; out-of-phase, 3.99 µV^2^) and incongruent (*Z* = − 2.51, *p* < 0.05; in-phase, 2.75 µV^2^; out-of-phase, 3.58 µV^2^) conditions. The differences between the two stimulation conditions in the neutral condition did not reach statistical significance (*Z* = − 1.69, *n.s.*). Regarding prestimulus alpha activity in the parieto-occipital region, there were no significant differences between the two stimulation conditions in the congruent (*Z* = 0.20, *n.s.*), neutral (*Z* = 0.11, *n.s.*), and incongruent (*Z* = − 0.14, *n.s.*) conditions. However, significantly enhanced prestimulus alpha power was detected over the frontocentral regions in the out-of-phase versus in-phase stimulation condition in the congruent (*Z* = − 2.23, *p* < 0.05; in-phase, 2.01 µV^2^; out-of-phase, 2.47 µV^2^) and incongruent (*Z* = − 2.23, *p* < 0.05; in-phase, 2.11 µV^2^; out-of-phase, 2.73 µV^2^) conditions (Fig. 8). The differences between the two stimulation conditions in the neutral condition (*Z* = − 1.60, *n.s.*) were not statistically significant. Since the spectral power showed significant differences between in-phase and out-of-phase tACS treatments, particularly in the theta and alpha bands (Fig. 8C), these observations were not simply due to the 1/f-like power spectral distribution of the EEG data [71].

## Discussion

Using phase-lagged tACS between two task-relevant brain regions, we observed improved behavioral performance and corresponding neurophysiological signatures during the inhibitory control task. Although previous non-invasive brain stimulation studies investigated the effects of tACS on inhibitory control by manipulating stimulation parameters, the utilization of temporal (or phasic) relationships between lDLPFC and dACC activity during inhibitory control is often overlooked. In the present study, out-of-phase stimulation between the lDLPFC and dACC yielded significantly faster task performance, significantly reduced N1 and N2 amplitudes, and significantly enhanced P1 and LSP amplitudes than in-phase stimulation, particularly in the incongruent condition. Additionally, out-of-phase stimulation resulted in significantly pronounced prestimulus frontocentral theta and alpha activity. Overall, our results demonstrate that phase-lagged tACS across task-relevant brain regions could effectively modulate the behavioral and neurophysiological aspects of inhibitory control.

Regarding the tACS-mediated alterations in ERP components, frontocentral N1 deflection exhibited reduced negative peak amplitudes following out-of-phase versus in-phase stimulation. N1 amplitude enhancement has been reported in relation to attentional facilitation directed toward the initial sensory processing of letter-component visual features such as line orientation and curvature [72–74]. Thus, the relatively enhanced N1 amplitude mediated by in-phase tACS promoted a more automatic but task-irrelevant word-reading process, consequently interrupting color-perceiving task performance. In this respect, out-of-phase stimulation might induce relatively less conflict between color and letter dimensions, facilitating the task-relevant color-perception process. A similar trend observed in the neutral condition, in which word reading was not prioritized, further supports this interpretation. The parieto-occipital P1 peaks were more prominent after out-of-phase than in-phase stimulation. This may reflect the facilitation of feature-specific top-down processing, specifically a boost in selective attention to color-based features [15, 75–78]. In effect, early bias toward task-relevant color processing was more pronounced in the out-of-phase stimulation sessions. 

The subsequent frontocentral N2 component exhibited reduced peak amplitudes during out-of-phase versus in-phase stimulation. With sources in the medial prefrontal regions, this reduction may indicate an out-of-phase tACS-mediated decrease in conflict activation or a lower demand to select task-relevant information before responding [34, 35, 79, 80]. This observation aligns with previous results showing that improved cognitive control is indexed by reduced dACC activation, quicker response times, and diminished N2 peak amplitudes [81–83]. Therefore, we can infer that top-down control functions were strengthened following out-of-phase stimulation, resulting in a more effective inhibition of distractors and a stronger bias toward task-relevant information. Finally, for the frontocentral LSP, we noted enhanced mean amplitudes during out-of-phase versus in-phase stimulation. This enhancement likely signifies the reinforced involvement of top-down executive processes and conflict resolution with sources in the lateral frontal cortices [28, 84, 85]. It is plausible that out-of-phase stimulation leads to an augmentation of top-down control, resulting in heightened selectivity toward task-relevant features and improved suppression of irrelevant information.

The EEG spectral analysis consistently supported the neuromodulatory effect of out-of-phase tACS on inhibitory control. Regarding EEG theta activity, out-of-phase tACS yielded significantly enhanced prestimulus theta power around the frontocentral region. This tACS-mediated augmentation likely indicates heightened anticipation and pre-activation of cognitive control for the impending task [68, 86–92]. Although previous studies linking theta power increase to preparation for subsequent conflict often presented cues before stimulus onset [87, 89–91], our experimental instructions required the participants to maintain the task strategy (i.e., recognizing colors of colored letters) throughout the task sessions. Furthermore, because the intertrial intervals in the present study were constant, there is likely an endogenously generated temporal anticipation of the upcoming trial [86, 92]. Taken together, out-of-phase tACS-mediated enhancement of top-down preparation may facilitate quicker adjustments when conflicts are encountered in a subsequent task interval, resulting in reduced reaction times. Accordingly, we also observed significantly prominent prestimulus alpha activity in the frontocentral region for out-of-phase versus in-phase stimulation. This increased prestimulus alpha activity implies improved inhibitory suppression of task-irrelevant processing associated with word reading, reflecting reinforced top-down regulatory control [69, 93–96]. Accordingly, the precise allocation of attention to task-relevant features appears to be facilitated by out-of-phase stimulation. Our ERP results also support this interpretation, demonstrating that preparatory attention influences early neural responses to stimulus features, consistent with the findings of previous studies [76, 95, 97–99].

Our neuromodulatory findings were consistent with those of previous neurodynamic studies on inhibitory control. For example, the control model [27] posits a temporal sequence in interference processing during a Stroop task. According to this model, DLPFC regions implement early top-down control by assigning greater importance to task-relevant sensory processing. Subsequently, cingulate regions select the appropriate information required to generate a response and provide feedback to the relevant areas [24, 25, 27]. The dual-network view of the attentional system [100] proposes a similar theory in which distinct networks collaborate in implementing top-down control. Specifically, the cingulo-opercular control system, encompassing the dACC, and the frontoparietal system, comprising the DLPFC, interact with unique roles [12, 100, 101]. Within this framework, a temporal flow exists and the interactions between the subregions of each network influence the task performance [101].

However, the present study has several limitations. First, owing to current technical limitations, the scalp-based tACS device was unable to effectively stimulate deep brain structures such as the dACC (Fig. 3C). Moreover, individual sensation thresholds of the input current contributed to further limitations in the stimulation power to the target areas. Because the mean simulation electric field (0.11 V/m) was below the minimum of 0.2 V/m that is required to modulate neurons in awake and behaving mammals [102, 103], a higher stimulation intensity would have improved the observed neuromodulatory effect. A recent neuromodulatory approach using temporal interference has demonstrated a non-invasive method for selectively stimulating deep brain structures [104, 105], which may provide further corroborating evidence for future studies. Second, the EEG theta frequency should be calculated under the no-tACS condition for the subsequent tACS resonating frequency, and a long experimental time (when both no-tACS and sham conditions are included) could induce fatigue in participants, leading to poor data quality. To consider this trade-off, the present study employed only the no-tACS condition, excluding the tACS-sham condition. Therefore, the present study focused on the tACS phase-dependent modulatory effect of in-phase (0°) and out-of-phase (180°) lags across the lDLPFC and dACC on subsequent behavioral and neurodynamic changes. Third, the in-phase and out-of-phase lag conditions were applied in a serial manner with a time gap of at least 10 min (Fig. 2B). Although there was a counter-balanced arrangement across the participants to cancel out the tACS after-effects, any possible tACS after-effects [106, 107] should be carefully considered when interpreting our findings.

## Conclusions

Our findings suggested that out-of-phase stimulation facilitated inhibitory control processing using optimally phase-lagged stimulation signals across task-relevant brain regions (i.e., the lDLPFC and dACC), thereby strengthening temporal neurodynamics across them. This neuromodulatory augmentation may effectively mitigate the Stroop interference effect and boost the inhibitory control processes. Notably, this effect remained unaffected by the speed-accuracy trade-off, resulting in improved reaction times without compromising task performance accuracy. It is probable that fine-tuning the phase lags of the tACS signals between the two task-relevant brain regions would provide further optimized neuromodulation. Additionally, spatially accurate neuroimaging approaches such as fMRI may reveal detailed tACS-mediated neurodynamics observed in task-relevant subcortical structures for inhibitory control. In summary, our observations provide promising evidence that out-of-phase tACS between the lDLPFC and the dACC effectively modulates selective attention and prestimulus top-down regulation, thereby facilitating inhibitory control performance. Our findings suggest that at least some of the variability in non-invasive brain stimulation effects may be attributed to temporal phase relationships across task-relevant brain regions, suggesting that this information should be considered in neuromodulatory paradigm designs.

### Electronic supplementary material

Below is the link to the electronic supplementary material.

Supplementary Material 1

Supplementary Material 2

## Data Availability

No datasets were generated or analysed during the current study.

## Abbreviations

BOLD: blood oxygenation level–dependent
dACC: dorsal anterior cingulate cortex
EEG: electroencephalography
ERP: event-related potential
FA: flip angle
fMRI: functional magnetic resonance imaging
lDLPFC: left dorsolateral prefrontal cortex
LSP: late sustained potentials
MRI: magnetic resonance imaging
tACS: transcranial alternating current stimulation
TE: echo time
TR: repetition time
