# Supplementary material for: Neuromodulation of inhibitory control using phase-lagged transcranial alternating current stimulation
Source: J Neuroeng Rehabil. 2024 May 30;21:93. doi: 10.1186/s12984-024-01385-y (PMC11138099; doi:10.1186/s12984-024-01385-y)
Supplement: Supplementary file 2 — Supplementary Material 2 [file 12984_2024_1385_MOESM2_ESM.docx]

**Supplementary Information** for

Neuromodulation of inhibitory control using phase-lagged transcranial alternating current stimulation

Yukyung Kim, Je-hyeop Lee, Je-choon Park, Jeongwook Kwon,

Hyoungkyu Kim, Jeehye Seo, Byoung-Kyong Min

Correspondence to: min_bk@korea.ac.kr


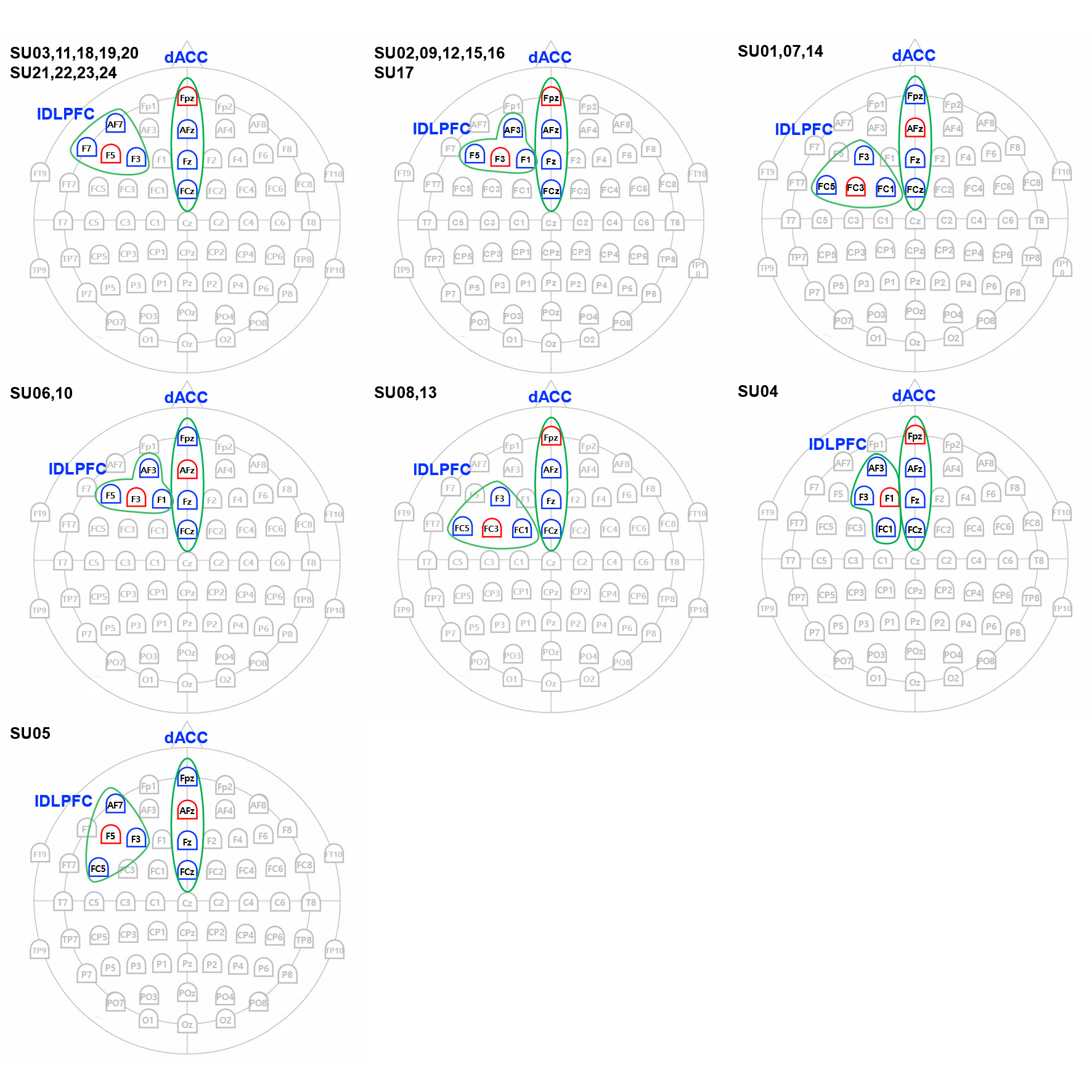
**Figure S1. Individual tACS channel montages.** Subject-specific optimized coordinates of stimulation target regions were computed based on individual T1 images for spatially accurate stimulation. Subject IDs were denoted with the initial “SU”. The red color represents stimulation channels, and the blue color represents the surrounding return channels.
